# Slowing down as we age: aging of the cardiac pacemaker’s neural control

**DOI:** 10.1007/s11357-021-00420-3

**Published:** 2021-07-22

**Authors:** Sabrina Choi, Matthias Baudot, Oscar Vivas, Claudia M. Moreno

**Affiliations:** Department of Physiology & Biophysics, University of Washington, Seattle, WA 98195 USA

**Keywords:** Heart rate, Cardiac pacemaker, Autonomic control, Cardiac aging

## Abstract

The cardiac pacemaker ignites and coordinates the contraction of the whole heart, uninterruptedly, throughout our entire life. Pacemaker rate is constantly tuned by the autonomous nervous system to maintain body homeostasis. Sympathetic and parasympathetic terminals act over the pacemaker cells as the accelerator and the brake pedals, increasing or reducing the firing rate of pacemaker cells to match physiological demands. Despite the remarkable reliability of this tissue, the pacemaker is not exempt from the detrimental effects of aging. Mammals experience a natural and continuous decrease in the pacemaker rate throughout the entire lifespan. Why the pacemaker rhythm slows with age is poorly understood. Neural control of the pacemaker is remodeled from birth to adulthood, with strong evidence of age-related dysfunction that leads to a downshift of the pacemaker. Such evidence includes remodeling of pacemaker tissue architecture, alterations in the innervation, changes in the sympathetic acceleration and the parasympathetic deceleration, and alterations in the responsiveness of pacemaker cells to adrenergic and cholinergic modulation. In this review, we revisit the main evidence on the neural control of the pacemaker at the tissue and cellular level and the effects of aging on shaping this neural control.

## Introduction

**Fig. 1:**
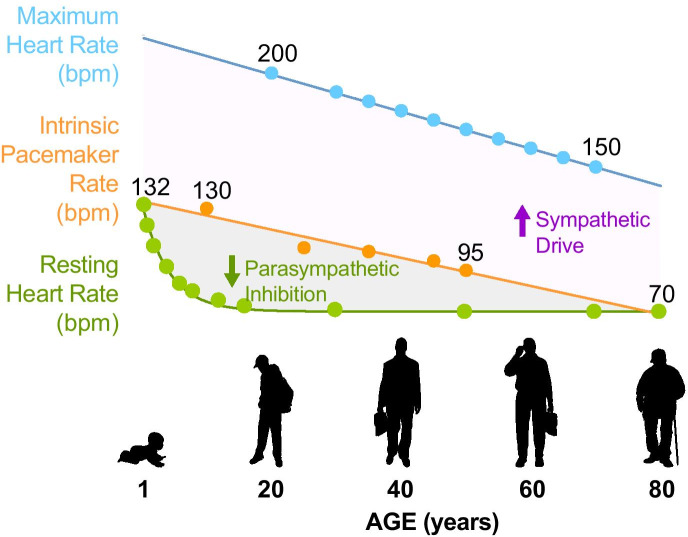
Comparison of maximum, intrinsic, and resting heart rates along human lifespan, illustrating the effect of age on the sympathetic and parasympathetic drive. The Intrinsic heart rate declines linearly from birth at a rate of ~ 0.8 bpm/year in humans [3]. Data to build the graph was obtained from Marcus et al. [9], Ostchega et al. [2], and the AHA

On average, the human heart beats 100,000 times a day. Every heartbeat starts with a subtle electrical spark inside the sinoatrial node, a small and highly specialized tissue located next to the right atrium also known as the cardiac pacemaker. The automaticity of the cardiac pacemaker relies on the unique ability of its cells to continuously generate action potentials, starting very early during embryonic development and working non-stop until the moment we die. Despite the remarkable reliability of this tissue, the pacemaker is not exempt from the detrimental effects of aging [1]. Mammals, including humans and mice, experience a natural and continuous decrease in the *intrinsic pacemaker rate* throughout their entire lifespan (Fig. 1) [2, 3]. The *intrinsic pacemaker rate* declines linearly from birth at a rate of ~ 0.8 bpm/year in humans and ~ 4 bpm/month in mice [3]. The slowdown of the intrinsic pacemaker rate is the main cause for the accompanying decline in *maximum heart rate*, playing a significant role in the loss of aerobic capacity in older adults. In pathological cases, this slowdown results in arrhythmia and sometimes sudden death as part of a group of idiopathic disorders known as Sick Sinus Syndrome [4]. This syndrome is the main cause for more than 600,000 artificial pacemaker implantations carried out annually in the world [5]. Hence, aging is the leading risk factor for heart pacemaker dysfunction, which justifies the urgency of understanding the pacemaker’s age-dependent decline. Although some of the mechanisms behind the pacemaker slowdown are being elucidated, there are still many unanswered questions.

Heart rate is finely tuned through neural control to maintain body homeostasis in constantly changing conditions. Slight internal changes in blood volume, CO_2_ levels, or pH trigger the activation of autonomic reflexes that directly modulate the intrinsic firing rate of pacemaker cells. In addition, pacemaker rate is abruptly changed in response to external stimuli as part of the fight-or-flight response activated under acute stress [1, 6]. Neural control of the pacemaker is achieved by a dense innervation, making the pacemaker the most innervated region of the heart [7]. The direct action of autonomic innervation on the firing of the pacemaker was first visualized by Otto Hutter and Wolfgang Trautwein in 1955, pioneers in the pacemaking field. Hutter and Trautwein photographed, for the first time ever, traces showing vagal stimulation depressed the pacemaker firing while stimulation with atropine accelerated it [8]. Since then, questions regarding pacemaker modulation, its pattern of innervation, and the disease-related changes in the neural control of the pacemaker have been further researched.

As shown in Fig. 1, heart rate varies with age, with a rapid decrease in resting heart rate from birth till late childhood, and a parallel linear decline in the maximum and the intrinsic pacemaker rate throughout the entire life [2, 9]. Age-associated changes in the resting heart rate depend mainly on the remodeling of the pacemaker’s neural control. During the first years after birth, there is a predominant sympathetic drive that maintains high resting rate values. However, throughout childhood, the parasympathetic tone increases and becomes predominant, setting lower resting rates that remain relatively stable throughout the lifespan.

In contrast, the age-associated linear decline in the intrinsic heart rate has been proposed to be caused by a combination of intrinsic and extrinsic mechanisms. Some of the identified intrinsic mechanisms behind the slowdown of the intrinsic pacemaker rate include (i) the reduction in the activation of the funny current (*I*_*f*_) carried by HCN channels, which is one of the main ionic currents sustaining the pacemaker automaticity [3, 10], (ii) a tissular remodeling driven by the loss of pacemaker cells [11–13], and (iii) a reduction in the sensitivity of pacemaker cells to adrenergic modulation. We refer the readers to a comprehensive review recently published by Peters et al. [1] covering these three main aspects. Extrinsic mechanisms linked to the age-associated pacemaker slowdown include an increase in tissue fibrosis and changes in the sympathetic/parasympathetic balance. There is evidence that aging causes an important remodeling in the sympathetic and parasympathetic modulation and the response of pacemaker cells to it. Despite the evident remodeling of the neural control of pacemaker function, little is known about how aging affects the innervation of the pacemaker and the neuro-pacemaker communication. Here, we will revisit the main evidence on the neural control of the pacemaker at the tissue and cellular levels and the effects of aging on shaping this neural control.

## Pacemaker anatomy and structural alterations during aging

**Fig. 2:**
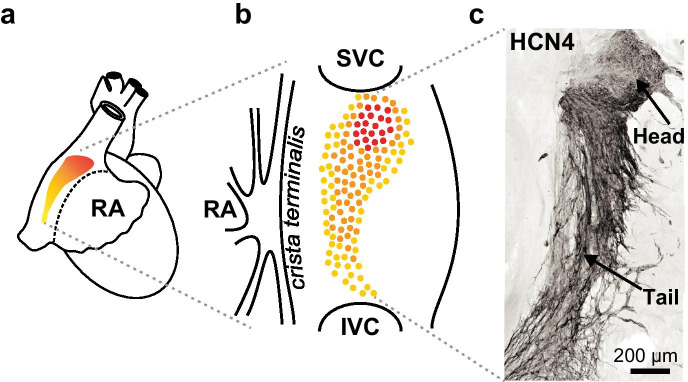
**a** Anatomical localization of the cardiac pacemaker next to the right atrium (RA). **b** Representation of the ventral view of the cardiac pacemaker delimited by the crista terminalis, the superior (SVC), and inferior vena cava (IVC). **c**. Representative super-resolution image of a cleared pacemaker from a 6-month-old mouse. Tissue was immunostained with the anti-HCN4 marker for pacemaker cells. Positive labeling is depicted in the inverted gray-scale image. The head and the tail regions of the pacemaker are indicated with the arrows

**Fig. 3:**
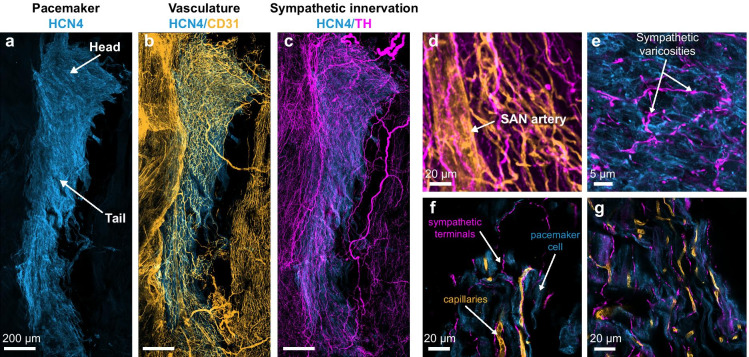
Representative 10X AiryScan super-resolution reconstruction of the mouse pacemaker. The pacemaker was isolated from a 4-month-old animal and immunostained against **a** the pacemaker marker HCN4 channel (blue), **b** the vascular marker CD31 (orange), and **c** the sympathetic marker TH (magenta). **d** Magnification of the sinoatrial node (SAN) artery (orange) and the sympathetic innervation of the vasculature (magenta). **e** Magnification to show the sympathetic axonal varicosities (magenta) in close contact with the pacemaker cells (blue). **f**–**g** 63X magnifications of two pacemaker regions to show the intricate contacts between the sympathetic terminals, the vascular tree, and the pacemaker cells

The cardiac pacemaker was anatomically identified in 1907 by Arthur Keith and Martin Flack. Only a select group of studies have such an impressive list of materials like that from Keith and Flack’s original paper [14]. By analyzing hearts from eels, salmon, frogs, lizards, turtles, moles, mice, cats, a kangaroo, a dolphin, humans, and even a whale, Keith and Flack identified inside the sino-auricular junction a conserved region of characteristic primitive wavy fibers which exhibited a close connection with the terminals innervating the heart. This led them to hypothesize that this region was the origin of the heart’s rhythm and the main target for the heart’s neural control; further studies would prove them right [15–17]. The pacemaker is located next to the right atrium and delimited to the left by the *crista terminalis*, to the bottom by the *inferior vena cava*, and to the top by the *superior vena cava* (Fig. 2). Size and position vary between species, but it is always delimited to the intercaval region [18]. Occupying only about 3% of the heart surface area [19], the pacemaker drives the contraction of the whole heart. Opposite to the large and highly organized cells that form the ventricles and atria, the pacemaker is formed by small wavy cells with poor content of myofibers immersed into a dense connective tissue network [20, 21]. The proper function of the pacemaker relies on its architecture [18]. Far from being a homogeneous structure, the pacemaker is formed by at least three morphologically different cell types, classified as spindle, elongated, and spider cell-types [22–24]. These cells are organized into a complex tridimensional structure divided into a *head* and a *tail* region (Figs. 2 and 3A). Although it is not yet clear how these three cellular subtypes organize inside the pacemaker or if they play different roles in the generation and conduction of the electrical signals, there is evidence in rabbits [25], dogs, and humans [26] that spindle cells inside the head of the pacemaker organize to form the *pacemaker lead.* The lead initiates the electrical signal that propagates through the pacemaker and travels along the electrical conduction system of the heart to trigger the contraction of the heart chambers [27]. The pacemaker *tail*, also called the peripheral area, is composed of less packed pacemaker cells running in a caudal direction (Fig. 2 and 3A). In some species, there is a gradual transition with pacemaker cells interspersed with atrial-like cells [18]. The tail plays an important role in the propagation and the exit of the electrical signal towards the right atrium. Electrical continuity is important for the conduction of the electrical signals. As such, pacemaker cells are connected through gap junctions formed by connexins 45, 40, and 30.2 to secure the rapid spread of the signal [18, 28–30]. Interestingly, cells at the center of the pacemaker do not connect to a vast extent, suggesting that they have a very specialized and efficient electrical coupling [31]. Given the high metabolic activity of this tissue, the pacemaker is highly vascularized, being supplied directly by the sinoatrial node artery and a vast arteriole and capillary network (Fig. 3B).

Architectural remodeling of the pacemaker tissue has been suggested as one of the factors leading to its dysfunction during aging. The main age-associated changes include changes in cell size, cell number, and fibrosis. Both cell atrophy [13] and hypertrophy [3, 32, 33] have been observed in old animals, so the association between cell size and dysfunction is still inconclusive. The number of pacemaker cells is inversely proportional to age [11, 34], and, in some cases, the amount of fibrotic tissue is positively correlated with age [11–13, 35]. However, fibrosis has also been observed in healthy individuals, suggesting that fibrosis does not necessarily lead to pacemaker dysfunction [35, 36]. Another common age-associated architectural change is the infiltration of fatty tissue, but its role in pacemaker dysfunction remains unknown [13]. Given the high metabolic demand of the pacemaker, another important and unexplored aspect is how aging affects the vascularization of the pacemaker.

## Autonomic heart rate modulation and aging

**Fig. 4:**
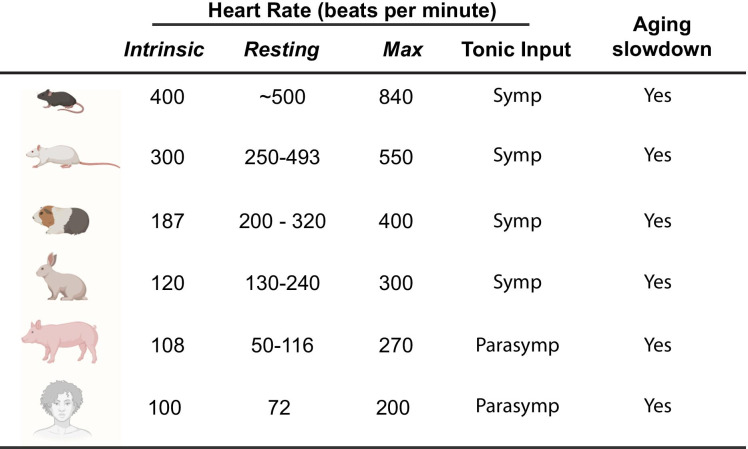
Species-specific heart rate ranges, autonomic tone, and evidence of age-associated slowdown. Data for the different species were obtained from [1, 122–138]. Figure created with Biorender

In order to understand the heart rate neural control, it helps to visualize the pacemaker as a car driving at a constant speed that can be overridden by pressing the accelerator or the brake. In the absence of any external input, the constant speed of the car is equivalent to the so-called *intrinsic heart rate*, which is dictated by how fast the pacemaker cells can fire action potentials. The accelerator pedal represents the sympathetic neurons innervating the pacemaker, which, by the release of noradrenaline, accelerates the pacemaker up to the *maximum heart rate*. The brake represents the parasympathetic nerve terminals, which, through the release of acetylcholine, decreases the *intrinsic rate* to reach the minimum level, commonly referred to as the *resting heart rate*. Humans have a tonic parasympathetic activity that explains why our *resting heart rate* is about 70 bpm, even though human pacemaker cells have an intrinsic rate of approximately 100 bpm. Intrinsic, resting, and maximum heart rate varies between species, but the age-associated progressive decline is a common denominator (Fig. 4).

### Pacemaker innervation

The pacemaker is the most innervated region of the heart, being innervated 3–4 times more than the surrounding atrial area [37, 38]. It receives sympathetic and parasympathetic terminals coming from neurons located in extracardiac and intracardiac ganglia [39]. While the extracardiac ganglia run parallel to the spinal cord and contain exclusively sympathetic neurons, the intracardiac ganglia are located in the interatrial surface [40] and contain a pool of afferent sensory neurons, interneurons, and efferent sympathetic and parasympathetic neurons [41]. We will not discuss in detail the morphology and function of the intracardiac nervous system here since a recent review on this topic by Fedele and Brand covers it extensively [42]. The dense nerve network interlaces with the complex pacemaker vascular bed and with the pacemaker cells (Fig. 3C and D). The sympathetic and parasympathetic axons have abundant varicosities (Fig. 3D), where the release of noradrenaline and acetylcholine occurs [43]. In contrast to most neurons, autonomic nerve terminals form “*en passant*” synapses, which lack any post-synaptic pacemaker counterpart [43]. However, there is still a debate on the exact nature of the neuro-pacemaker communication interphase.

The pacemaker innervation is remodeled from birth to adulthood. In infants, sympathetic innervation is predominant, which correlates with the higher heart rates observed at that stage (Fig. 1). Parasympathetic nerve density increases during childhood until reaching a similar abundance to that seen for sympathetic nerves during adulthood [38]. Aging causes a reduction in both sympathetic and parasympathetic nerve density of the pacemaker [38]. Loss of sympathetic innervation has also been observed in other target tissues, including the pineal gland [44] and the spleen [45]. The remaining sympathetic nerves in these organs are swollen and contain aggregates and degenerating organelles. In the pineal gland, for example, the ratio of dystrophic axons to target pinealocytes increased by 30-fold in 5-months-old to 23-months-old male rats and by 200-fold in female rats. Interestingly, gender differences have been observed at the functional and morphological levels in the pineal gland [44]. More studies are needed to understand how aging affects the neuroanatomy of the sympathetic terminals directly in the pacemaker. Regarding the parasympathetic innervation, there is evidence of a loss of parasympathetic preganglionic neurons in the dorsal vagal nucleus and nucleus ambiguus on the right side of the medulla, which are the nuclei innervating the heart [46]. Although the number of intracardiac parasympathetic neurons does not change with age [47], a 45% reduction in the synaptic-like contacts in old animals has been reported [48]. More studies are needed to understand the effects of aging on the neuroanatomy and communication of the parasympathetic network with the sympathetic terminals, the vasculature, and the pacemaker cells.

### Pacemaker’s sympathetic acceleration

**Fig. 5:**
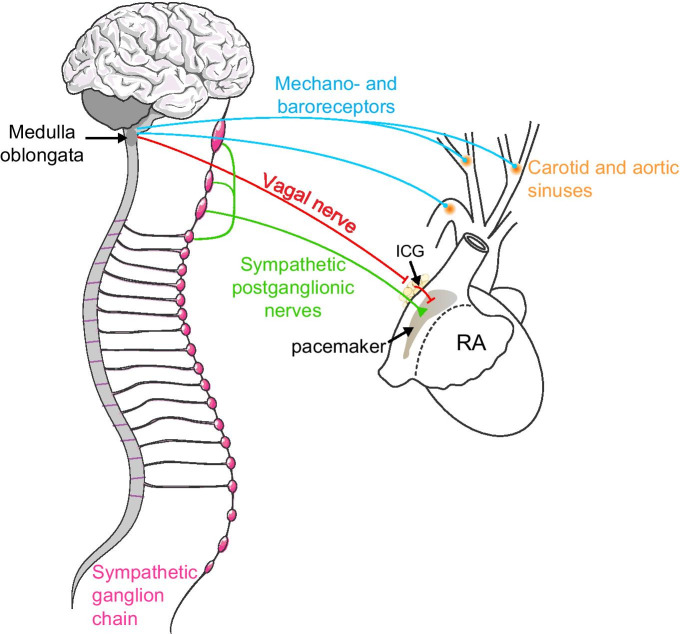
Representation of the cardio-motor pathway controlling heart rate. Mechano- and baroreceptors in carotid and aortic sinuses sense changes in pH, blood levels, and blood volume, and transmit information to nuclei in the medulla oblongata. Sympathetic innervation on the pacemaker originates from postganglionic neurons in the sympathetic ganglion chain. The parasympathetic pathway via the vagus nerve synapses with intracardiac ganglia (ICG) on the interatrial region to finally innervate the pacemaker

Heart rate is accelerated by the direct action of noradrenaline released from sympathetic neurons that innervate the cardiac pacemaker. This positive chronotropic modulation is controlled by the cardio motor pathway (Fig. 5), starting with the activation of sensory neurons coming from extracardiac ganglia. These mechano- and chemoreceptors sense various stimuli, including changes in pH, CO_2_, blood levels, and blood volume. Information is conveyed to excitatory neurons in the nucleus *tractus solitarius* located in the brain stem. From here, inhibitory and excitatory neurons synapse to the caudal and rostral ventrolateral medulla to control preganglionic excitatory neurons located in the intermediolateral nucleus at the spinal cord. The ultimate target is the postganglionic neurons residing in the sympathetic cervical and stellate ganglia [49, 50]. These postganglionic neurons directly innervate the pacemaker cells and release the noradrenaline that accelerates the pacemaker intrinsic firing rate.

With age, the sympathetic nervous system becomes hyperactive. Microneurographic recordings have shown that the characteristic bursting activity of sympathetic nerves increases with age, from 40 bursts/100 heartbeats in 20-year-old men to 65 bursts/100 heartbeats in 65-year-old men [51–53]. The age-dependent increase in sympathetic activity is more prominent in women, being 2.5-fold more pronounced than in men [54]. The increased firing activity of sympathetic nerves results in increased basal levels of noradrenaline in plasma. Noradrenaline levels increase with age, from 200 pg/ml in 20-year-old men to 400 pg/ml in 65-year-old men [53, 55–57]. In conclusion, aging leads to increased activity of the sympathetic nervous system, resulting in more noradrenaline released from the nerve terminals into target organs, including the pacemaker. Since the increase in the release of noradrenaline from the nerve terminals does not stop the slowdown of the pacemaker, it has been suggested that sympathetic hyperactivity is a response to the loss of pacemaker sensitivity to the adrenergic drive. The reason for this hyposensitivity might be a combination of reduced innervation and changes in the molecular mechanisms that sense the adrenergic signaling inside the pacemaker cells [58, 59].

### Pacemaker’s parasympathetic deceleration

In an antagonistic manner, heart rate is decelerated in response to the activation of the parasympathetic nervous system (Fig. 5) [60]. Opposite stimuli from those activating the sympathetic pathway also come from extracardiac mechano- and chemoreceptors, activating parasympathetic preganglionic neurons located in the medulla oblongata, specifically at the dorsal vagal nucleus and the nucleus ambiguous, from which the parasympathetic vagus nerve arises. The axons traveling through the vagus nerve directly innervate parasympathetic postganglionic neurons located at the intracardiac ganglia on the surface of the interatrial region. Parasympathetic terminals form synapses directly with sympathetic neurons to inhibit noradrenaline release through the release of acetylcholine. In addition, acetylcholine also acts over pacemaker cells to reduce their intrinsic firing rate.

Aging has an effect on pacemaker parasympathetic modulation. A reduction in the parasympathetic tone occurs with age and it has been suggested to compensate for the pacemaker intrinsic slowdown [61, 62]. Heart rate variability, which is largely accepted to reflect the parasympathetic modulation of the heart, decreases by more than 60% from 20-year-old to 60-year-old subjects [63–65], suggesting a loss of parasympathetic tone with age. Interestingly, a biological model has been proposed to explain the decrease in heart rate variability. The Neuron-Immune-Senescence Integrative Model links brain degeneration of the central parasympathetic regions with a decreased activity in the peripheral parasympathetic nerves that innervate targets including the heart, leading to a reduced heart rate variability [66]. Interestingly, this model goes beyond the effect on the heart and suggests that reduced activity of the parasympathetic nervous system results in increased production of pro-inflammatory cytokines IL-6 and TNFα, leading to cellular senescence and affecting the whole organism and, therefore, influencing multiple disorders associated with aging. We consider it noteworthy to point out that parasympathetic activity predominates in younger subjects, while in older ones sympathetic and parasympathetic tones are equilibrated by a simultaneous hyperactivation of sympathetic neurons and the hypoactivation of the parasympathetic ones [67]. It is expected that these concomitant changes in the autonomic inputs have a profound effect on the modulation of heart rhythm as we age.

## Aging and pacemaker acceleration and deceleration at the molecular level

The autonomic nervous system controls pacemaker action potential rate through a cascade effect of secondary pathways where the modulation of ion channels and calcium handling are the final pieces. The automaticity of the pacemaker is driven by diastolic depolarizing mechanisms that include the activation of HCN channels at hyperpolarized voltages, the spontaneous release of calcium from the sarcoendoplasmic reticulum through ryanodine receptors (RyR) coupled with L-type channel activation [68, 69], and the increase in the activity of the calcium sodium exchanger (NCX) [60, 70, 71]. The initial activation of the HCN channels together with the local rise in calcium depolarizes the cell enough to recruit Ca_V_3.1 (T-type) and Ca_V_1.3 (L-type) calcium channels; this further depolarization brings the membrane potential to the threshold to activate Ca_V_1.2 L-type calcium channels, which together with Ca_V_1.3 channels sustain the spike of the pacemaker action potential [72, 73]. Repolarization is mediated by the activation of potassium channels, mainly the delayed rectifier K + (KCNQ1) [1] and the ERG channel [74, 75].

At the molecular level, sympathetic acceleration starts by the binding of noradrenaline released from the sympathetic fibers to beta-adrenergic receptors (β-AR) located in the plasma membrane of pacemaker cells [60]. β-ARs are Gα_s_-coupled receptors. Their activation triggers the dissociation of the Gα_s_ subunit that activates adenylyl cyclase (AC) to increase the production of cAMP [70]. It is important to note that both, β1-AR and β2-AR, are expressed in a 1:1 ratio in pacemaker cells, indicating high expression of β2-AR in these cells compared to ventricular cardiomyocytes [76]. While β1-AR has been the most researched, β2-AR is also crucial to study as it activates the same stimulatory pathway and may have additional functional responses [76–78]. Evidence showing that β1-AR stimulation raises global cAMP levels while β2-AR stimulation raises local cAMP levels further suggests that these two pathways might have differential roles in pacemaker rate modulation [79].

The cAMP elevation caused by the activation of the adrenergic stimulation increases pacemaker firing rate through direct and indirect mechanisms [70]. cAMP directly binds to HCN channels, increasing their open probability and shifting their activation curve to less hyperpolarized voltages [18, 71, 80]. cAMP also acts indirectly, as a second messenger, activating protein kinase A (PKA), which phosphorylates and modulates many target proteins such as the α subunit of L-type calcium channels, increasing their open probability [71, 81]; β-AR receptors, decreasing desensitization; the sarcoplasmic reticulum handling protein phospholamban, increasing the rate of calcium accumulation and total storage capacity of the sarcoplasmic reticulum [82, 83]; and RyR, increasing their open probability [84]. Another important target is the phosphorylation of Ser-27 in the KCNQ1 channel, which increases the repolarization velocity, shortening the pacemaker action potential duration [85, 86].

Although phosphorylation of L-type calcium channels was proposed as the main mechanism to explain the adrenergic calcium channel facilitation [87], recent studies in ventricular cardiomyocytes have proposed an alternative mechanism for this modulation. A recent study by Liu et al. [88] showed that beta-adrenergic regulation persists on transgenic murine hearts expressing PKA phosphorylation-site-deficient mutant Ca_V_1.2 channels. Therefore, they propose a new mechanism that involves the PKA-mediated phosphorylation of the calcium channel inhibitor protein Rad that is tonically inhibiting calcium channels under resting conditions. Another mechanism involving the fusion of endosomes containing Ca_V_1.2 in response to β-AR stimulated PKA phosphorylation to increase the availability of channels at the plasma membrane has been recently proposed for ventricular cardiomyocytes by Del Villar et al. [81]. However, whether these alternative mechanisms are also present in pacemaker cells is still unknown.

On the other hand, pacemaker deceleration at the molecular level is mediated by the release of acetylcholine from parasympathetic fibers. Acetylcholine binds to type 2 muscarinic (M2R) receptors coupled to Gα_i_ proteins that inactivate the adenylyl cyclase pathway, reducing cAMP formation and counterbalancing sympathetic drive [60]. The reduction in cAMP production causes a shift in the activation-curve of HCN channels to more negative voltages and a reduction in RyR open probability as a result of the lack of PKA-mediated phosphorylation [89, 90]. Additionally, activation of G-protein regulated potassium channels by Gβγsubunits results in a deep hyperpolarization that accounts for 50% of the heart rate reduction [71, 91]. Acetylcholine also activates directly *I*_KACh_ currents that results in a more negative maximal diastolic potential, decreasing the pacemaker rate [18, 71]. Altogether, the changes induced by acetylcholine result in a negative chronotropic effect [60, 71, 92].

Aging also affects the neural control of the pacemaker at the molecular level. Pacemaker cells from old animals retain the capacity to be accelerated by adrenergic stimulation in a similar percentage to cells from young animals [3]. Accordingly, aging does not change either the transcription levels of beta-adrenergic receptors [93, 94] or the adrenergic-facilitation of calcium and HCN channels [3]. However, since old cells have a slower intrinsic firing rate, they fail to reach the same maximum firing rate as that observed in young animals, even under saturating concentrations of the beta-adrenergic agonist isoproterenol [3, 95]. Infusion of isoproterenol in patients under autonomic blockade was 39% less efficient in accelerating heart rate in 65-year-old subjects when compared to young 25-year-old subjects [95]. This effect was also observed in ex vivo isolated pacemakers, where around 5 times more isoproterenol was needed to increase the rate by 50% [96]. In a very interesting study by Sharpe et al., it has been shown that directly activating AC with forskolin and at the same time inhibiting phosphodiesterase with IBMX to increase total cAMP cytoplasmic concentration failed to accelerate old pacemaker cells firing rate to the same level as young cells. However, a high concentration of exogenous cAMP completely abolished the effects of aging on the slowdown of the intrinsic firing rate, restoring action potential firing rate and *I*_f_ absolute activation to the same levels observed in young mice [10], suggesting that all the machinery downstream AC can be potentiated to the same levels in old pacemaker cells. Other reported changes that can account for the intrinsic slowdown and the cap on the adrenergic response include a reduction in the expression of Ca_V_1.2, HCN4, Na_V_1.5, and several K^+^ channels [94, 97, 98] and a persistent hyperpolarized shift in the activation of HCN channels [10].

A reduced responsivity to the parasympathetic drive has been also observed in ex vivo isolated pacemakers, where around 6 times more carbachol, a cholinergic agonist, was needed to increase the beating intervals by 50% [96]. A reduction in the expression of muscarinic receptors type 2 by 45% in old pacemaker tissue has been associated with this reduction in responsivity [93]. A reduced responsivity to the parasympathetic drive can also be attributable to a common mechanism such as a reduction of cAMP/PKA sensitivity [99] or an age-reduced sensitivity to phosphodiesterase inhibition [96]. Parasympathetic input then appears to be attenuated in old animals, generating an abnormal sympathovagal balance with age [65].

## Global cardiac aging and pacemaker dysfunction

Although the mechanisms behind the age-associated dysfunction of the cardiac pacemaker are still being elucidated, it is believed to be a multifactorial process. Besides pacemaker failure, aging is also a risk factor for the onset of other cardiomyopathies, including heart failure and atrial fibrillation [100, 101]. How these age-associated alterations of ventricular and atrial function impact the pacemaker is not known. In ventricular and atrial myocytes, aging is linked to cellular senescence, mitochondrial dysfunction, and calcium mishandling. Cardiac aging drives a loss of ventricular and atrial cells and a compensatory cellular hypertrophy [102, 103]. Cardiac senescence in heart failure and atrial fibrillation is accompanied by a general decline in mitochondrial function, characterized by clonal expansion of dysfunctional mitochondria, increased production of reactive oxygen species, and dysregulation of mitochondrial fusion and fission [104]. Calcium mishandling is perhaps one of the most prominent age-associated changes in ventricular and atrial cells. Aging causes alterations in the diastolic levels of cytoplasmic calcium, the sarcoplasmic reticulum calcium content, and the calcium leakage from the stores, resulting in poor excitation–contraction coupling [105].

Furthermore, while there is a close relation between the incidence of heart failure, atrial fibrillation, and pacemaker dysfunction [106], the causality of one over the other is still not clear. Heart failure is commonly associated with atrial dysfunction. The increase in left ventricular end diastolic pressure observed in heart failure patients causes an increase in the intra-atrial pressure and atrial stretch, which results in atrial electrical remodeling and atrial dilatation [107]. It has been proposed that cellular and tissue alterations caused by heart failure can also cause electrical and structural remodeling of the pacemaker [108]; the identified mechanisms so far include a decrease in the HCN4 and sodium currents [109, 110]. However, it is not clear how malfunction of the ventricular chambers can result in ionic changes of pacemaker cells. The relationship between atrial fibrillation and pacemaker dysfunction seems more reciprocate, with the appearance of atrial fibrillation being able to cause pacemaker anomalies and vice versa [111, 112].

Some of the age-associated ventricular alterations can be reverted using the mTOR inhibitor, rapamycin. Rapamycin treatment in old animals decreases ventricular hypertrophy and increases ejection fraction [113] and diastolic function [114]. Nevertheless, changes in heart rate have not been reported after rapamycin treatment. Whether rapamycin has the potential of ameliorating age-associated dysfunctions of the cardiac pacemaker remains elusive.

## Summary of the effects of age on the function and the neural control of the pacemaker

**Fig. 6:**
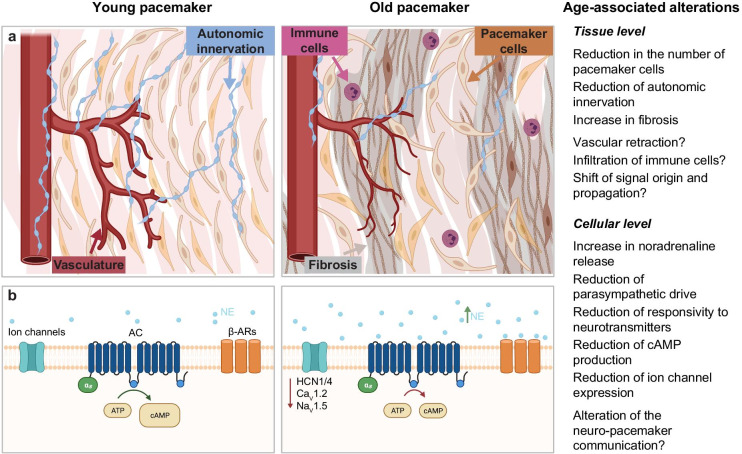
Schematic summarizing the known and unexplored effects of age on the function and neural control of the pacemaker, emphasizing alterations at the **a** tissue and **b** cellular level. Figure created with Biorender

Aging is accompanied by a natural slowdown of the pacemaker rate. In some cases, the pacemaker’s slowdown becomes pathological, leading to the onset of Sick Sinus Syndrome, a group of idiopathic diseases that account for more than half of about 250,000 artificial pacemakers implanted every year in the USA. Besides the implantation of artificial devices, there is no alternative treatment for this dysfunction. Advancing our understanding of the age-associated changes in the pacemaker may inform potential novel interventions to diagnose and treat this disease. Although the mechanisms behind the dysfunction of the pacemaker are not well understood, here, we summarize the main age-associated changes on the architecture and neural control of the pacemaker that can play an important role in this phenomenon (Fig. 6). Aging is associated with the following:

- A reduction in the number of pacemaker cells.
- A reduction in the autonomic innervation.
- An increase in fibrosis. Although the role of fibrosis on the dysfunction of the pacemaker has been controversial, given the importance of cell-to-cell connectivity for the propagation of the electrical signal, it is likely that the age-associated increase in fibrosis plays an important role on reshaping pacemaker function.
- A global increase in the sympathetic drive, resulting in the release of more noradrenaline.
- A reduced responsivity of the pacemaker to adrenergic stimulation.
- A reduction in the capacity of pacemaker cells to reach optimal cytoplasmic levels of cAMP.
- Alterations in the expression and function of a variety of ion channels that are important for the automaticity and that are targets of the adrenergic modulation, including HCN, L-type, T-type, and K^+^ channels.
- A reduction in the parasympathetic drive. These functional alterations result in an overall autonomic imbalance biased toward a higher sympathetic acceleration in the elderly.

## Perspectives

The prevalence and clinical implications of the age-associated dysfunction of the pacemaker justifies the urgency to identify the molecular mechanisms underlying it. Besides the need of defining the mechanisms for the intrinsic changes, alterations in other components of the pacemaker need more attention, including changes in the vascularization, in the generation and propagation of the electrical signal, and in the neuro-pacemaker communication.

Vascular dysfunction plays an important role in organ pathology. The brain is perhaps the most studied example, where age causes structural and functional alterations of the vascular bed leading to dysregulation of cerebral blood flow, ischemia, and impaired clearance of metabolic byproducts [115]. The pacemaker is also a heavily vascularized tissue, a requisite to meet its high metabolic demands and to allow the fast control of heart rate by the action of bloodstream circulating adrenaline. Therefore, alterations in blood supply could be an important stressor that might contribute to the dysfunction of the pacemaker in the elderly.

Where in the pacemaker the action potential is originated and how it propagates inside the tissue to exit toward the atrium is fundamental for setting heart rate. The leading pacemaker site is not static, rather, in a phenomenon known as pacemaker shift, it moves from the center of the pacemaker head to the periphery or even to the tail depending on external inputs [116]. Adrenergic or cholinergic stimulation [32, 92], changes in extracellular ionic concentration, or the action of drugs can cause the shift of the pacemaker to the region with higher or lower sensitivity to the stimulus, depending on the excitatory or inhibitory nature of it [117]. Pacemaker shift denotes a heterogeneity in the cell identity or the molecular mechanisms that sense different stimuli. It is necessary to study how aging affects this heterogeneity to fully understand pacemaker dysfunction.

The classical view of the communication between autonomic terminals and pacemaker cells depicts the release of neurotransmitters from varicosities and the free diffusion nearby pacemaker cells [37]. However, there is evidence supporting the existence of neuro-cardiac synapses [118]. Testing the effect of aging on this neuro-pacemaker communication will require the refinement of techniques to measure the release, binding, and diffusion of noradrenaline and acetylcholine in the intact pacemaker.

Finally, another unexplored area is how cellular senescence and age-associated exacerbation of the immune response can affect pacemaker function. Growing evidence positions senescence and inflammation as important drivers for cardiac dysfunction [119–121], but how they specifically affect cardiac pacemaker function remains elusive.

## Abbreviations

AC: Adenylyl cyclase
β-AR: Beta adrenergic receptor
cAMP: Cyclic adenosine monophosphate
HCN: Hyperpolarization-activated cyclic nucleotide-gated channels
*I*_f_: Funny current (HCN)
IVC: Inferior vena cava
M2R: Muscarinic receptor type 2
NCX: Calcium-sodium exchanger
PKA: Protein kinase A
RA: Right atrium
RyR: Ryanodine receptor
SAN: Sinoatrial node
SVC: Superior vena cava
